# Up-regulated LINC01234 promotes non-small-cell lung cancer cell metastasis by activating VAV3 and repressing BTG2 expression

**DOI:** 10.1186/s13045-019-0842-2

**Published:** 2020-01-20

**Authors:** Zhenyao Chen, Xin Chen, Binbin Lu, Yu Gu, Qinnan Chen, Tianyao Lei, Fengqi Nie, Jingyao Gu, Jiali Huang, Chenchen Wei, Ming Sun, Zhaoxia Wang

**Affiliations:** 1 Cancer Medical Center, The Second Affiliated Hospital of Nanjing Medical University, Nanjing, Jiangsu People’s Republic of China 210011; 2 Faculty of Mathematics, University of Waterloo, Waterloo, Canada; 3 Department of Bioinformatics and Computational Biology, UT MD Anderson Cancer Center, Houston, TX 77030 USA

**Keywords:** NSCLC, Metastasis, LINC01234, VAV3, BTG2

## Abstract

**Background:**

Long noncoding RNAs (lncRNAs) are known to regulate tumorigenesis and cancer progression, but their contributions to non-small-cell lung cancer (NSCLC) metastasis remain poorly understood. Our previous and other studies have revealed the involvement of upregulated LINC01234 in regulating gastric cancer and colon cancer cells proliferation, and we aimed to investigate whether LINC01234 overexpression also contribute to cancer cells metastasis in this study.

**Methods:**

We collect the NSCLC tissues and adjacent non-tumor tissues and analyzed expression levels of LINC01234 by quantitative reverse-transcription PCR. LINC01234 were knocked down by using siRNAs or shRNAs, and overexpressed by transfection with overexpression vector; RNA levels of miRNA were downregulated or upregulated with inhibitors or mimics. Transwell assays were used to evaluate cell migration and invasive ability; in vivo metastasis experiments were performed to investigate the effect of LINC01234 on NSCLC cells metastasis. Luciferase reporter, RIP, and ChIP assays were used to determine the regulation of LINC01234 on its targets.

**Results:**

LINC01234 expression is increased in NSCLC tissues, and its upregulation is associated with metastasis and shorter survival in NSCLC. Downregulation of LINC01234 impairs cell migration and invasion in vitro, and inhibits cells metastasis in vivo by acting as a competing endogenous RNA for the miR-340-5p and miR-27b-3p. LINC01234 also interacts with the RNA-binding proteins LSD1 and EZH2, leading to histone modification and transcriptional repression of the anti-proliferative genes BTG2.

**Conclusions:**

Taken together, our findings identify two oncogenic regulatory axes in NSCLC centering on LINC01234: one involving miR-340-5p/miR-27b-3p in the cytoplasm and the second involving EZH2, LSD1, and BTG2 in the nucleus. Our study indicates that these genes may be targeted to reduce or prevent NSCLC metastasis.

## Introduction

Lung cancer is the leading cause of cancer-related death in men world-wide [1]. About 80% of all lung cancer diagnoses are non-small-cell lung cancers (NSCLCs), the major subtypes being lung adenocarcinoma and lung squamous cell carcinoma. Despite rapid advances in diagnostic techniques, molecular-targeted drugs, and immune checkpoint therapy, the 5-year overall survival (OS) time of NSCLC patients remains less than 15% due to lots of patients diagnosed with NSCLC had distant metastases which has been a consistent problem in tumor therapy. Several genetic alterations have been reported to be “drivers” of NSCLC progression, including mutations in epidermal growth factor receptor and mesenchymal–epidermal transition-related genes. Nevertheless, the mechanisms underlying NSCLC progression are not well understood [2–5]. Therefore, exploration of the molecular mechanisms involved in the progression of NSCLC is critical to improving treatment and the rates of patient survival.

Advanced sequencing techniques and bioinformatics methods have revealed that protein-coding genes only occupy a small proportion (~ 2%) of the whole human genome, while the rest encodes a large number of noncoding RNAs (ncRNAs) including small ncRNAs, pseudogenes, and long noncoding RNAs (lncRNAs) [6–8]. Accumulating evidences have uncovered that lncRNAs are pivotal regulators that affect tumorigenesis and cancer progression through affecting almost every aspect of cancer cells behaviors, including cell growth, apoptosis, autophagy, invasion, and metastasis. For example, Wang etc. reported that SATB2-AS1 suppressed colorectal carcinoma aggressiveness through recruiting p300 to the SATB2 promoter and upregulating its expression, which subsequently inhibited Snail transcription and epithelial-to-mesenchymal transition [9]. Our previous studies revealed that overexpression of the lncRNA HOXA11-antisense (AS) facilitates gastric metastasis by interacting with HuR to regulate β-catenin mRNA stability in gastric cancer [10]. As a result, lncRNAs have been highlighted as novel players in tumor metastasis. However, only a small part of these lncRNAs function and underlying mechanisms in cancer progression were clarified, while most of which remains largely unknown.

We speculate that there are still a great number of lncRNAs that are closely related to NSCLC metastasis that have not been explored. In our previous study, we identified a gastric cancer-associated lncRNA LINC01234, which promoted gastric tumorigenesis via sponging miR-204-5p to regulate CBFB expression [11]. Interestingly, a recent study found that LINC01234 could also regulate cell invasion in esophageal cancer [12]. In this study, we analyzed differentially expressed lncRNAs between NSCLC tissues with metastasis and those without metastasis and found that LINC01234 was significantly increased in the metastatic specimen. We further investigated the functional roles and characterized the molecular mechanisms of LINC01234 in NSCLC progression.

## Materials and methods

### NSCLC sample collection and cell lines

A total of 45 paired NSCLC specimens and adjacent noncancerous tissues were obtained from 45 patients with a histopathological diagnosis of NSCLC who underwent surgery at the Second Affiliated Hospital of Nanjing Medical University between 2010 and 2013. These patients did not receive chemotherapy or radiotherapy prior to surgery. Tissue samples were immediately snap-frozen in liquid nitrogen and stored at − 80 °C until required. This study was approved by the Research Ethics Committee of Nanjing Medical University and informed consent was obtained from all patients.

Four NSCLC adenocarcinoma cell lines (A549, SPC-A1, H1299, and PC9), a NSCLC squamous carcinoma cell line (H226), and a normal human bronchial epithelial cell line (16HBE) were purchased from the Institute of Biochemistry and Cell Biology of the Chinese Academy of Sciences (Shanghai, China). A549, H1299, and H226 cells were maintained in RPMI 1640 basic medium, and SPC-A1, PC9, and 16HBE cells were maintained in DMEM medium with 10% fetal bovine serum, 100 U/ml penicillin, and 100 μg/ml streptomycin (Invitrogen, Carlsbad, CA, USA). All cells were maintained in a humidified 5% CO_2_ atmosphere at 37 °C.

### RNA extraction and quantitative reverse transcription-PCR assays

Total RNA was extracted from tissues or cultured cells using TRIzol reagent (Invitrogen) according to the manufacturer’s instructions. Aliquots of 1 μg RNA were reverse transcribed to cDNA in a final volume of 20 μl under standard conditions using a PrimeScript RT Reagent Kit (TaKara, Dalian, China). Real-time PCR analyses were performed with SYBR Premix Ex Taq (Takara) on an Applied Biosystems 7500 Real-Time PCR System. Expression of LINC01234 and other genes was normalized to that of glyceraldehyde-3-phosphate dehydrogenase (GAPDH) using the relative threshold cycle method, and then converted to fold changes. Specific primers are listed in Additional file 1: Table S1. Primers for miR-27b-3p, miR-340-5p, and U6 were purchased from GeneCopoeia (Rockville, MD, USA). Our quantitative reverse transcription-PCR (qRT-PCR) results were analyzed and expressed relative to threshold cycle (CT) values, and then converted to fold changes.

### Plasmid construction and cell transfection

The LINC01234 sequence was synthesized according to the full-length cDNA of human LINC01234 and BTG2 sequences were synthesized according to its coding sequences. All sequences were cloned into the expression vector pCDNA3.1 (Invitrogen). Control and LINC01234-targeting short hairpin RNAs (shRNAs) were purchased from Invitrogen and inserted into the pLKO.1 vector. All final constructs were verified by sequencing. Plasmids were purified using DNA Midiprep Kits (Qiagen, Valencia, CA) and transfected into NSCLC cells using X-treme GENE HP DNA transfection reagent (Roche, Basel, Switzerland). Three LINC01234-targeting small interfering RNAs (siRNAs) obtained from Invitrogen and other gene-targeting siRNAs, miRNA mimics, and miRNA inhibitors (Genepharma, Shanghai, Chian) were transfected into NSCLC cells using Lipofectamine 2000 (Invitrogen) according to the manufacturer’s instructions. Nucleotide sequences for the siRNAs and shRNAs are listed in Additional file 1: Table S1. Cells were harvested 48 h after transfection and analyzed as indicated for the individual experiments.

### Luciferase reporter assays

Online bioinformatics databases DIANA Tools (http://carolina.imis.athena-innovation.gr/diana_tools/web/index.php) and miRbase (http://www.mirbase.org/) were used to predict potential miRNA-binding sites in LINC01234. The putative binding sequences were synthesized, inserted into the pGL3-Basic luciferase reporter vector (Promega), and verified by sequencing. Vectors were transfected into human HEK293T cells for 48 h, and luciferase activity was then measured using a Dual Luciferase Kit (Promega), according to the manufacturer’s instructions. The data are presented as relative firefly luciferase activity normalized to the *Renilla* luciferase activity. All experiments were performed three times.

### Cell migration and invasion assays

For cell migration and invasion assays, cells were collected at 48 h post-transfection, and 5 × 10^4^ (for migration assay) or 1 × 10^5^ (for invasion assay) cells in serum-free medium were placed into the upper chamber of an insert (8-μm pore size; Millipore, Billerica, MA, USA). Medium containing 10% fetal bovine serum was added to the lower chamber. After incubation for 24 h, cells in the upper chamber were removed with cotton swabs, and the cells on the lower membrane surface were fixed and stained with 0.5% crystal violet solution. Experiments were independently performed three times.

### Animal experiments

All protocols were approved by the Committee on the Ethics of Animal Experiments of the Nanjing Medical University and were carried out in strict accordance with the recommendations of the Guide for the Care and Use of Laboratory Animals of the National Institutes of Health. For metastasis assays, SPC-A1 and A549 cells stably transfected with control shRNA or sh-LINC01234 (3 × 10^6^) were injected intravenously via the tail vein. Eight weeks post-injection, the mice were sacrificed and the lungs were removed and photographed. Tumors visible on the lung surface were counted, and the lungs were then stored in formalin.

### Subcellular fractionation

Cytoplasmic and nuclear RNA were isolated and purified from NSCLC cells using a PARIS Kit (Life Technologies), according to the manufacturer’s instructions.

### RNA immunoprecipitation

RNA immunoprecipitation (RIP) assays were performed using an EZ Magna RIP kit (Millipore) using the manufacturer’s protocol. A549 and SPC-A1 cells were lysed in complete lysis buffer, and the extracts were incubated with magnetic beads conjugated with the appropriate specific antibodies or control IgGs (Millipore) for 3–6 h at 4 °C. The beads were washed, incubated with proteinase K to remove proteins, and the purified RNA was eluted and analyzed for the presence of LINC01234 by qRT-PCR. Details of the antibodies and primers are given in Additional file 1: Table S1.

### RNA pull-down assays

LINC01234 or control RNAs were transcribed in vitro from pcDNA3.1-LINC01234 using T7 RNA polymerase (Ambion Life) and purified using an RNeasy Mini Kit (Qiagen). One aliquot of transcribed LINC01234 RNA was biotinylated with a Biotin RNA Labeling Mix (Ambion Life). Positive control, negative control, nonbiotinylated, and biotinylated RNAs were incubated with A549 cell lysates. Streptavidin-conjugated magnetic beads were then added and the samples were incubated at room temperature. The beads were then washed, and the eluted proteins were examined by western blot analysis.

### Chromatin immunoprecipitation assays

Chromatin immunoprecipitation (ChIP) assays were performed using a MagnaChIP Kit (Millipore) according to the manufacturer’s instructions, as described previously [13].

### Western blot analysis

A549 and SPC-A1 cells were lysed with RIPA extraction reagent (Beyotime) supplemented with a protease inhibitor cocktail (Roche). Proteins in cell lysates were separated by 10% sodium dodecyl sulfate-polyacrylamide gel electrophoresis and transferred to 0.22 μm polyvinylidene fluoride membranes (Millipore). Membranes were probed with specific antibodies using standard methods. Specific protein bands were detected by incubation with ECL chromogenic substrate and quantified by densitometry (Quantity One software; Bio-Rad, Hercules, CA, USA). Antibodies against E-cadherin, N-cadherin, Vimentin, and GAPDH (1:1000) were purchased from Cell Signaling Technology; antibodies against VAV3, EZH2, LSD1, Ago2, and HuR were purchased from Millipore; antibody against BTG2 was purchased from Absin. GAPDH was probed as an internal control. Antibodies are listed in Additional file 1: Table S1.

### Statistical analysis

Statistical analyses were performed using SPSS 20.0 (IBM, Armonk, NY, USA) and Prism software (GraphPad, La Jolla, CA, USA). LncRNA expression levels in primary solid tumors and normal solid tissue samples were compared using the Mann–Whitney *U* test. For the remaining assays, differences between groups were assessed by paired, two-tailed Student’s *t* test, Wilcoxon’s test, or χ^2^ test, as appropriate. Spearman’s correlation analysis was used to calculate the correlations between clinical factors and LINC01234, miR-27b-3p, miR-340-5p, BTG2, and VAV3 expression. All tests were two-sided, and a *P* value < 0.05 was considered statistically significant.

## Results

### LINC01234 expression is upregulated in NSCLC and correlates with poor prognosis

**Fig. 1:**
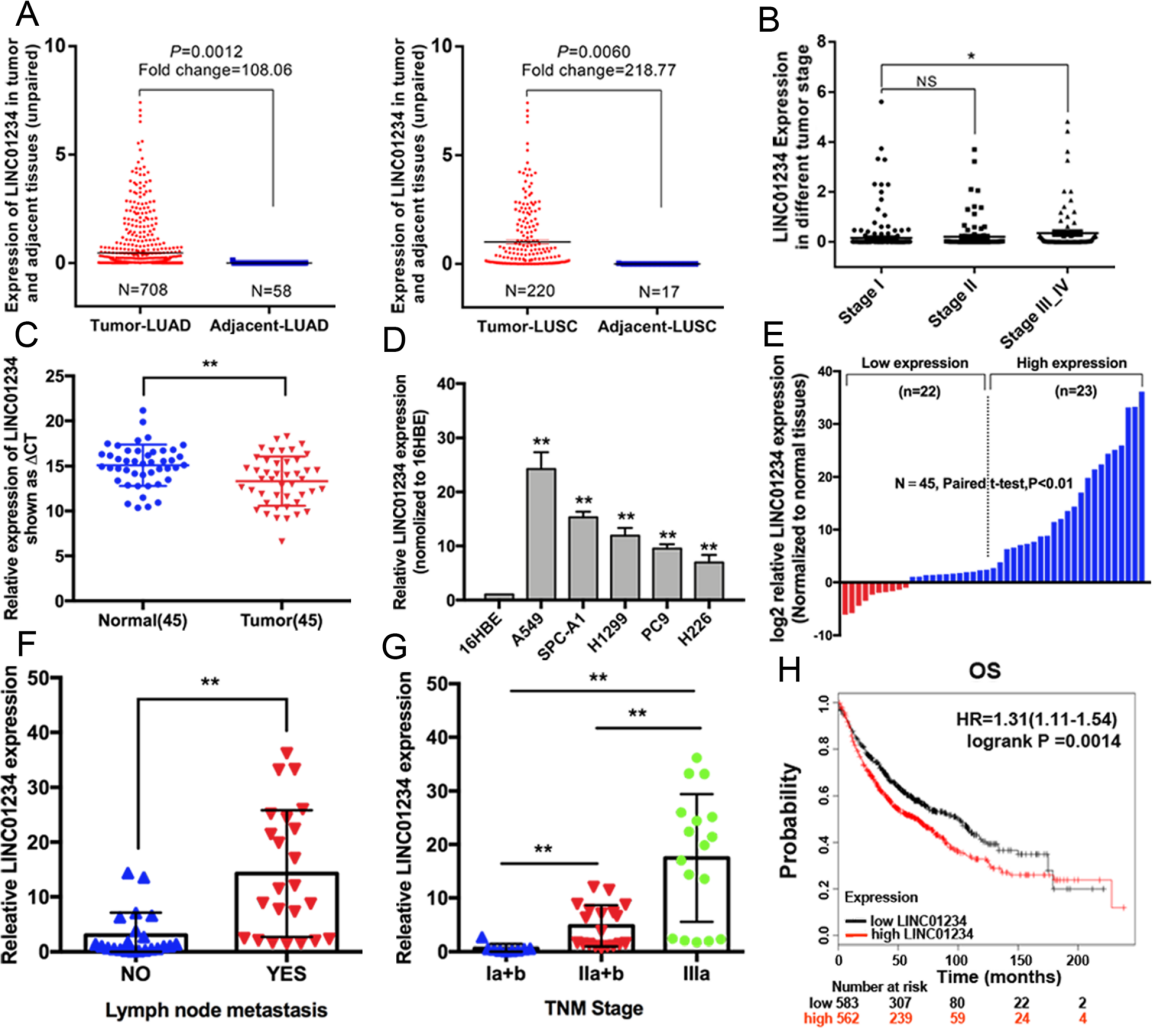
LINC01234 is overexpressed in NSCLC and its clinical significance. **a** Data mining of altered lncRNA expression in TCGA NSCLC sequencing data (LUAD and LUSC). **b** Data mining of correlation between LINC01234 expression and lung adenocarcinoma stage in TCGA dataset. **c** qRT-PCR analysis of LINC01234 expression in 45 pairs of NSCLC tissues and adjacent non-tumor lung tissues. **d** qRT-PCR analysis of LINC01234 expression in 16HBE cells and five NSCLC cell lines. **e** Forty-five NSCLC tumor tissues were divided into two groups (high and low LINC01234 expression) based on the median value. **f**, **g** Relationship between LINC01234 expression and clinicopathological features of patients with NSCLC. **h** Kaplan–Meier survival plots of OS based on LINC01234 expression in lung cancer patients (*n* = 1928). *P < 0.05, **P < 0.01

We first analyzed lung adenocarcinoma and lung squamous cell carcinoma RNA sequencing datasets from TCGA and found LINC01234 was upregulated in NSCLC tissues compared with adjacent tissues (Fig. 1a). In addition, we found a significant correlation between LINC01234 expression and lung adenocarcinoma stage from TCGA dataset (Fig. 1b). Furthermore, we examined the expression level of LINC01234 in NSCLC tissues and cell lines. qRT-PCR analysis of 45 paired NSCLC and adjacent normal tissues indicated significant upregulation of LINC01234 (fold-change > 1; *P* < 0.01) in 78% (35/45) of cancerous tissues compared with normal tissues (Fig. 1c). Results also indicated that LINC01234 showed increased expression in NSCLC cell lines in comparison to normal bronchial epithelial cells (Fig. 1d).

Next, we explored the relationship between LINC01234 expression and clinicopathological features of patients with NSCLC. For this, the 45 NSCLC tumor tissues were assigned to two groups (high and low LINC01234 expression) based on the median value (Fig. 1e). Higher LINC01234 expression level was also significantly correlated with tumor size (Chi-square test, *P* = 0.042), TNM stage (Chi-square test, *P* = 0.015), and lymph node metastasis (Chi-square test, *P* = 0.011) in NSCLC patients, but not with other factors, including sex and age (Fig. 1f, g, Additional file 1: Table S2). Univariate Cox regression analyses identified histologic grade, lymphatic metastasis, TNM stage, and LINC01234 expression level as prognostic factors. Other clinicopathologic features such as sex and age were not statistically significant prognosis factors. Multivariate Cox regression analyses showed that LINC01234 expression level were independent prognostic factors for NSCLC patients (Additional file 1: Table S3). Kaplan–Meier survival analysis showed that the OS rates were lower for the high vs. low LINC01234 expression groups, which was supported by Kaplan–Meier Plotter analysis (www.kmplot.com) [14] (Fig. 1h).

### LINC01234 modulates NSCLC cell migration and invasion in vitro and metastasis in vivo

**Fig. 2:**
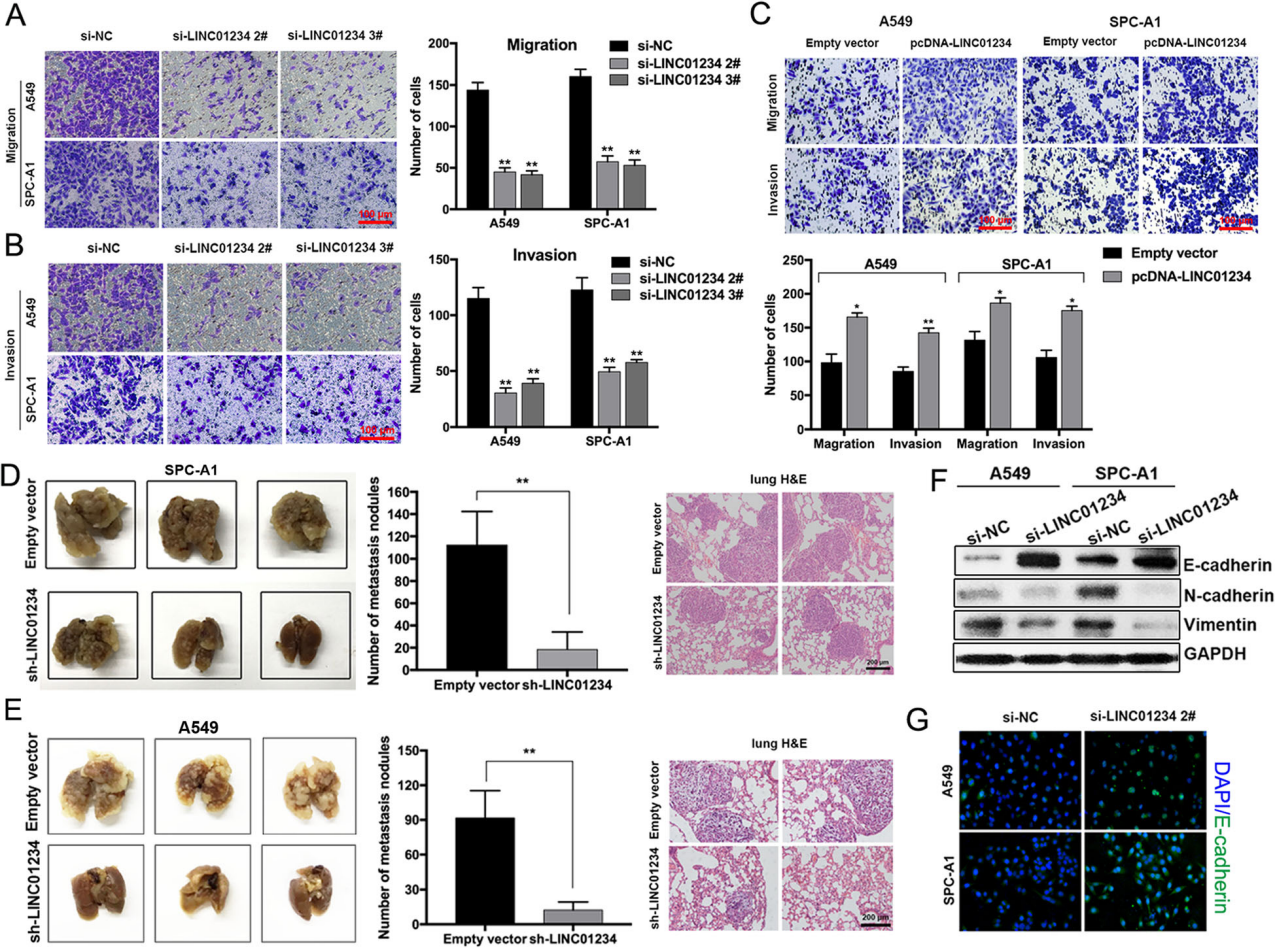
LINC01234 promote NSCLC cells metastasis in vitro and in vivo. **a–c** Transwell assays of LINC01234-depleted (**a**, **b**) and LINC01234-overexpressing (**c**) NSCLC cells. **d**, **e** Experimental nude mouse model of metastasis of SPC-A1 and A549 cells stably transfected with LINC01234. Representative images of mouse lungs and number of visible tumor nodules on lung surfaces. Representative images of lungs and H&E-stained lung sections. **f** Western blot analysis of E-cadherin, N-cadherin, and Vimentin expression in NSCLC cells transfected with LINC01234 siRNAs. **g** Immunofluorescence analysis of E-cadherin in NSCLC cells depleted of LINC01234. *P < 0.05, **P < 0.01

To assess the mechanisms by which LINC01234 promotes the progression of NSCLC, we firstly performed transwell migration and invasion assays. We found that LINC01234 knockdown significantly decreased the migration and invasion ability of A549 and SPC-A1 cells compared with cells expressing si-NC, while overexpression of LINC01234 promoted A549 and SPC-A1 cells migration and invasion (Fig. 2a–c). To validate these results in vivo, we examined the metastatic potential of A549 and SPC-A1 cells stably transfected with empty vector or sh-LINC01234 after injection into nude mice. Consistent with the in vitro analyses, LINC01234 knockdown reduced the number of metastatic lung nodules compared with the control group. H&E staining of excised lung sections confirmed the lower frequency of metastases in the LINC01234-deleted tumors (Fig. 2d, e). However, we did not find metastases in the liver, kidney, intestine, spleen, and other organs (Additional file 1: Figure S1A). Interestingly, expression of the epithelial–mesenchymal transition (EMT) marker E-cadherin was increased, whereas N-cadherin and Vimentin were decreased in LINC01234 downregulated cells (Fig. 2f). In addition, E-cadherin protein levels were also found to be upregulated in A549 and SPC-A1 cells by immunofluorescence assay (Fig. 2g). These in vivo data therefore complement the results of the in vitro functional studies and establish LINC01234 as a regulator of NSCLC metastasis.

### LINC01234 interacts with RNA-binding proteins to modulate target gene expression in NSCLC cells

**Fig. 3:**
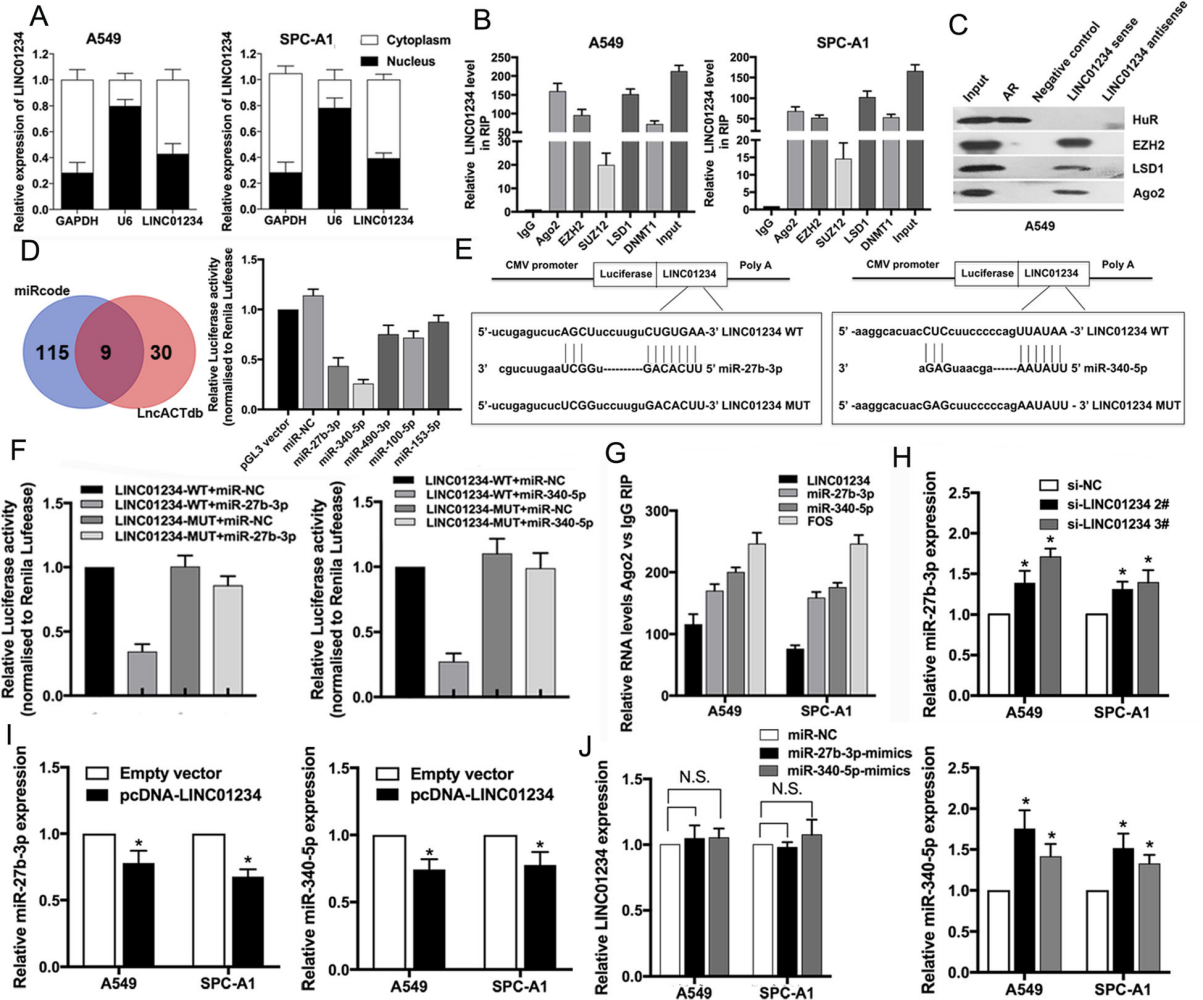
LINC01234 interacts with RNA-binding proteins and functions as a ceRNA for miR-27b-3p and miR-340-5p in NSCLC cells. **a** qRT-PCR analysis of the subcellular localization of LINC01234 in A549 and SPC-A1 cells. **b** RIP assays showing LINC01234 co-immunoprecipitation with EZH2, LSD1, and Ago2 from A549 and SPC-A1 cells. **c** RNA-pull-down assays showing EZH2, LSD1 and Ago2 binding to desthiobiotinylated LINC012345 from A549 cells. Androgen receptor (AR) RNA binding to HuR was analyzed as a positive control. **d** Predicted miRNA-binding sites in LINC01234 identified using online bioinformatics databases (left). Validation of miRNA sponges for LINC01234 by luciferase reporter assays. Luciferase activity was normalized to *Renilla* activity (right). **e** Schematic diagram of the predicted binding sites for miR-27b-3p and miR-340-5p in LINC01234. **f** Validation of miR-27b-3p and miR-340-5p sponges for LINC01234 by luciferase reporter assays. Luciferase activity was normalized to Renilla activity. **g** Immunoprecipitation of Ago2 and qPCR of associated RNAs (LINC01234, miR-27b-3p, and miR-340-5p). **h**, **i** Relative expression of miR-340-5p and miR-27b-3p in NSCLC cells overexpressing or depleted of LINC01234. **j** Relative expression of LINC01234 in NSCLC cells transfected with miR-340-5p or miR-27b-3p mimics. *P < 0.05, **P < 0.01

To better understand the underlying mechanism of LINC01234 in NSCLC, we examined its distribution in NSCLC cells via subcellular fractionation. Interestingly, qRT-PCR analysis of RNA purified from nuclear and cytoplasmic cellular fractions revealed that LINC01234 RNA was localized both in nucleus and cytoplasm (Fig. 3a). We also performed ISH to detect the distribution of LINC01234 in NSCLC tissues. The results showed that LINC01234 was expressed both in nucleus and cytoplasm in NSCLC tissues (Additional file 1: Figure S1B). Multiple studies have indicated that lncRNAs could regulate gene expression at transcription level by interacting with RNA binding proteins, such as EZH2, SUZ12, and LSD1. lncRNAs could also affect gene expression at post-transcription level by binding with Ago2. RIP assays confirmed the presence of LINC01234 in EZH2, LSD1, and Ago2 immunoprecipitates from A549 and SPC-A1 cell lysates (Fig. 3b). We next performed RNA pull-down assays to identify LINC01234-associated proteins. We found that biotinylated LINC01234 RNA, but not labeled negative control or antisense RNAs, bound to EZH2, LSD1, and Ago2 (Fig. 3c). Collectively, these data demonstrate that LINC01234 binds to EZH2, LSD1, and Ago2 proteins in NSCLC cells.

### LINC01234 functions as a ceRNA for miR-27b-3p and miR-340-5p in NSCLC cells

Emerging evidence suggests that some lncRNAs regulate target gene expression by functioning as ceRNAs, or sponges, for miRNAs, thereby preventing miRNA-mediated regulation of target mRNAs. Interestingly, one of the LINC01234-binding proteins identified above is Ago2, a component of the RNA-induced silencing complex involved in miRNA-mediated repression of mRNAs. Therefore, we investigated the possibility that LINC01234 may also function as a ceRNA. To test this hypothesis, we searched the online bioinformatics databases LncACTdb and miRcode for putative miRNA-binding sites in the LINC01234 sequence. Both databases identified nine putative sites (Fig. 3d), from which we analyzed five with known tumor suppressor functions (miR-27b-3p, miR-340-5p, miR-490-3p, miR-100-5p, and miR-153-5p). Luciferase reporter assays showed that co-transfection with miR-27b-3p and miR-340-5p significantly decreased the luciferase activity of HEK293T cells harboring a LINC01234-driven reporter construct compared with control miRNAs (Fig. 3d). Mutation of the putative miR-27b-3p and miR-340-5p binding sites in LINC01234 abolished their ability to suppress luciferase expression, confirming that the miRNAs specifically interact with LINC01234 (Fig. 3e, f). Consistent with this, Ago2 immunoprecipitates from A549 and SPC-A1 cell lysates were enriched in LINC01234, miR-27b-3p, and miR-340-5p compared with control IgG immunoprecipitates (Fig. 3g). Finally, miR-27b-3p and miR-340-5p levels in A549 and SPC-A1 cells were significantly increased and decreased by LINC01234 depletion and overexpression, respectively (Fig. 3h, i), whereas overexpression of miR-27b-3p or miR-340-5p had no effect on LINC01234 levels (Fig. 3j). These data indicate that LINC01234 acts as a sponge to directly interact with miR-27b-3p and miR-340-5p.

### VAV3 is a target of miR-27b-3p and miR-340-5p and is indirectly regulated by LINC01234

**Fig. 4:**
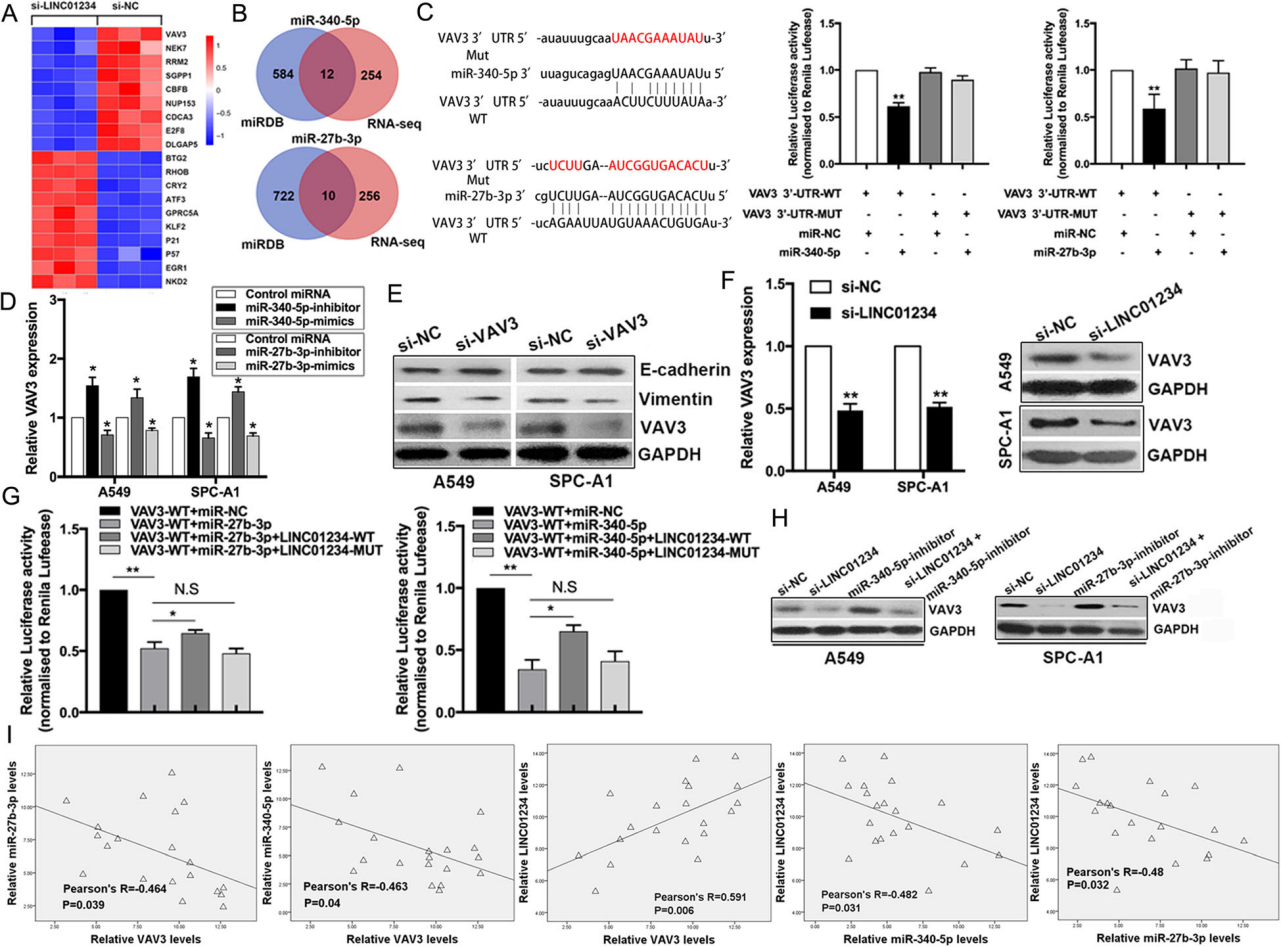
VAV3 is a key target of miR-340-5p and miR-27b-3p in NSCLC cells. **a** Hierarchically clustered heatmap of mRNAs upregulated and downregulated in A549 cells after transfection of LINC01234 or control (NC) siRNAs. **b** Genes regulated by miR-27b-3p, miR-340-5p, and LINC01234 based on miRDB database and RNA-seq data analysis. **c** Predicted binding sites for miR-340-5p and miR-27b-3p in VAV3 mRNA. Luciferase activities were measured in NSCLC cells co-transfected with luciferase reporter containing VAV3 wild-type or mutant and the mimics of miR-340-5p and miR-27b-3p. **d** VAV3 mRNA levels in NSCLC cells depleted of miR-340-5p or miR-27b-3p. **e** Western blot analysis of E-cadherin, Vimentin and VAV3 expression in NSCLC cells transfected with VAV3 siRNAs. **f** Western blot and qRT-PCR analysis of VAV3 expression in LINC01234-depleted NSCLC cells. **g** Luciferase activities were measured in NSCLC cells co-transfected with luciferase reporter containing LINC01234 wild-type or mutant type, VAV3 wild-type, mimics of miR-340-5p and miR-27b-3p. **h** VAV3 protein levels in NSCLC cells co-transfected with LINC01234 siRNA and miR-340-5p or miR-27b-3p inhibitors. (**I**) Correlations between VAV3, miR-340-5p and miR-27b-3p expression. Correlation analysis of LINC01234 expression and VAV3 expression. *P < 0.05, **P < 0.01

To identify LINC01234-regulated target genes in NSCLC in an unbiased manner, we performed RNA-seq to compare the gene expression profiles of A549 cells expressing LINC01234 siRNA or si-NC (Fig. 4a). To identify target genes regulated by both miRNAs and LINC01234, we performed an integrative analysis of the miRDB database and the gene expression profiles of LINC01234-depleted and control A549 cells. Among the 266 genes significantly downregulated by LINC01234 silencing (log_2_fold-change > 1 and *P* < 0.05), 12 and 10 were target genes of miR-340-5p and miR-27b-3p, respectively. Interestingly, VAV3 was identified as a target gene for both miRNAs (Fig. 4b). We validated the LINC01234, miR-27b-3p, miR-340-5p, and VAV3 network using luciferase reporter assays. We cloned the wild-type 3′-untranslated region (3′UTR) of VAV3 (wt-VAV3) or one carrying mutations in the miR-27b-3p/miR-340-5p-binding sites (mut-VAV3) into the luciferase vector and co-transfected them with control miRNA or miR-27b-3p/miR-340-5p mimics into HEK293T cells. We found that miR-27b-3p and miR-340-5p significantly decreased luciferase activity driven by wt-VAV3 but not by mut-VAV3 (Fig. 4c), indicating that VAV3 mRNA levels are directly regulated by miR-27b-3p/miR-340-5p in NSCLC cells. Consistent with this, VAV3 mRNA levels in NSCLC cells were reduced or elevated by expression of miR-27b-3p/miR-340-5p mimics or inhibitors, respectively (Fig. 4d). Additionally, E-cadherin was increased, whereas Vimentin was decreased, in VAV3 knockdown cells (Fig. 4e). Knockdown of LINC01234 significantly reduced VAV3 mRNA and protein levels in A549 and SPC-A1 cells, which is consistent with the loss of miR-27b-3p and miR-340-5p sponging (Fig. 4f). In luciferase reporter assays, miR-27b-3p and miR-340-5p-mediated suppression of wt-VAV3-driven luciferase activity was partly reversed by co-expression of the wild-type LINC01234 sequence, but not by LINC01234 mutated in the miR-27b-3p/miR-340-5p binding sites (Fig. 4g). Finally, the suppression of VAV3 protein levels induced by LINC01234 silencing was effectively reversed by co-expression of miR-27b-3p/miR-340-5p inhibitors in NSCLC cells (Fig. 4h). Moreover, the expression levels of miR-27b-3p/miR-340-5p in NSCLC tissues were negatively related with VAV3 expression, while LINC01234 expression was positively related to VAV3 expression. Additionally, miR-27b-3p/miR-340-5p were negatively correlated with LINC01234 (Fig. 4i). Collectively, these data indicate that LINC01234 modulates the expression of VAV3 by acting as a ceRNA for miR-27b-3p and miR-340-5p in NSCLC cells.

### miR-27b-3p, miR-340-5p, and VAV3 were involved in NSCLC progression

**Fig. 5:**
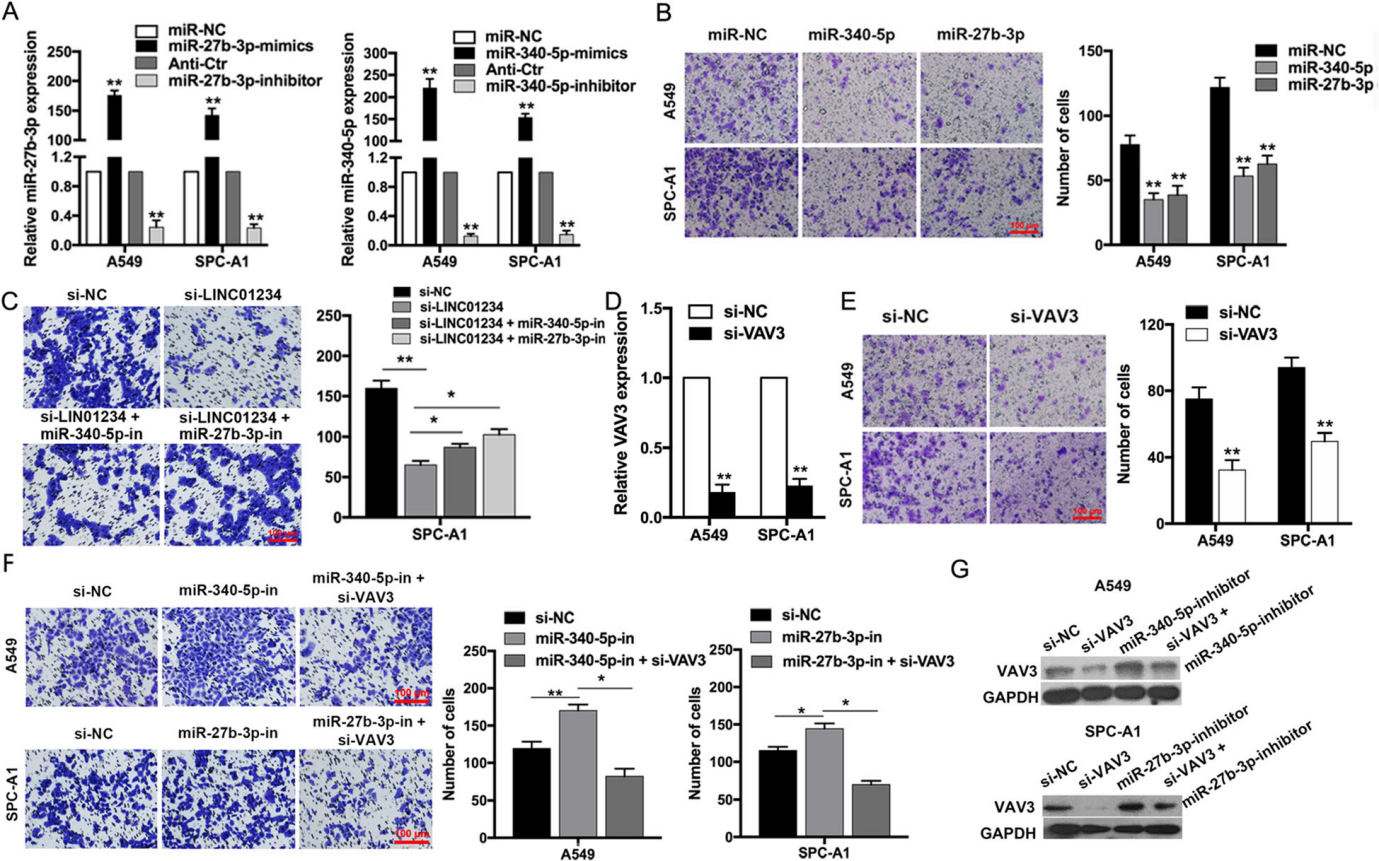
miR-27b-3p, miR-340-5p and VAV3 were participated in NSCLC cells metastasis. **a** Relative expression of miR-340-5p and miR-27b-3p in NSCLC cells transfected with miR-340-5p and miR-27b-3p mimics or inhibitors. **b** Transwell assays of the proliferative and invasive capacity of A549 and SPC-A1 cells overexpressing miR-340-5p or miR-27b-3p. **c** Transwell assays of SPC-A1 cells co-transfected with si-LINC01234, miR-340-5p inhibitors, or miR-27b-3p inhibitors. **d** qRT-PCR of VAV3 mRNA levels in NSCLC cells transfected with VAV3 siRNA. **e** Transwell assays of VAV3-depleted A549 and SPC-A1 cells. **f** Transwell assays of NSCLC cells expressing miR-340-5p or miR-27b-3p inhibitors, and partial rescue by VAV3 inhibition. **g** VAV3 protein levels in NSCLC cells co-transfected with VAV3 siRNA and miR-340-5p or miR-27b-3p inhibitors. *P < 0.05, **P < 0.01

To determine whether miR-27b-3p and miR-340-5p have tumor suppressor activities, we transfected A549 and SPC-A1 cells with miRNA mimics or inhibitors and examined the effects on cell function (Fig. 5a). Expression of miR-27b-3p and miR-340-5p mimics significantly reduced cell invasion (Fig. 5b). Moreover, co-transfection with miR-27b-3p/miR-340-5p inhibitors partially reversed the inhibition of cell invasion induced by LINC01234 knockdown (Fig. 5c). Next, we silenced VAV3 expression in A549 and SPC-A1 cells by transfection with VAV3 siRNA, which was confirmed to be effective by qRT-PCR (Fig. 5d). Inhibition of VAV3 significantly inhibited NSCLC cell invasion (Fig. 5e). In addition, co-transfection with VAV3 siRNA partially reversed the promotion of A549 and SPC-A1 cell invasion induced by transfection with miR-27b-3p/miR-340-5p inhibitors (Fig. 5f). The increasing in VAV3 protein levels in miR-27b-3p/miR-340-5p inhibitor transfected A549 and SPC-A1 cells was also partly rescued by co-transfection with VAV3 siRNA (Fig. 5g). Collectively, these results suggest that miR-27b-3p, miR-340-5p, and VAV3 are involved in mediating the functional effects of LINC01234 in NSCLC cells.

### BTG2 is critical downstream targets of LINC01234

**Fig. 6:**
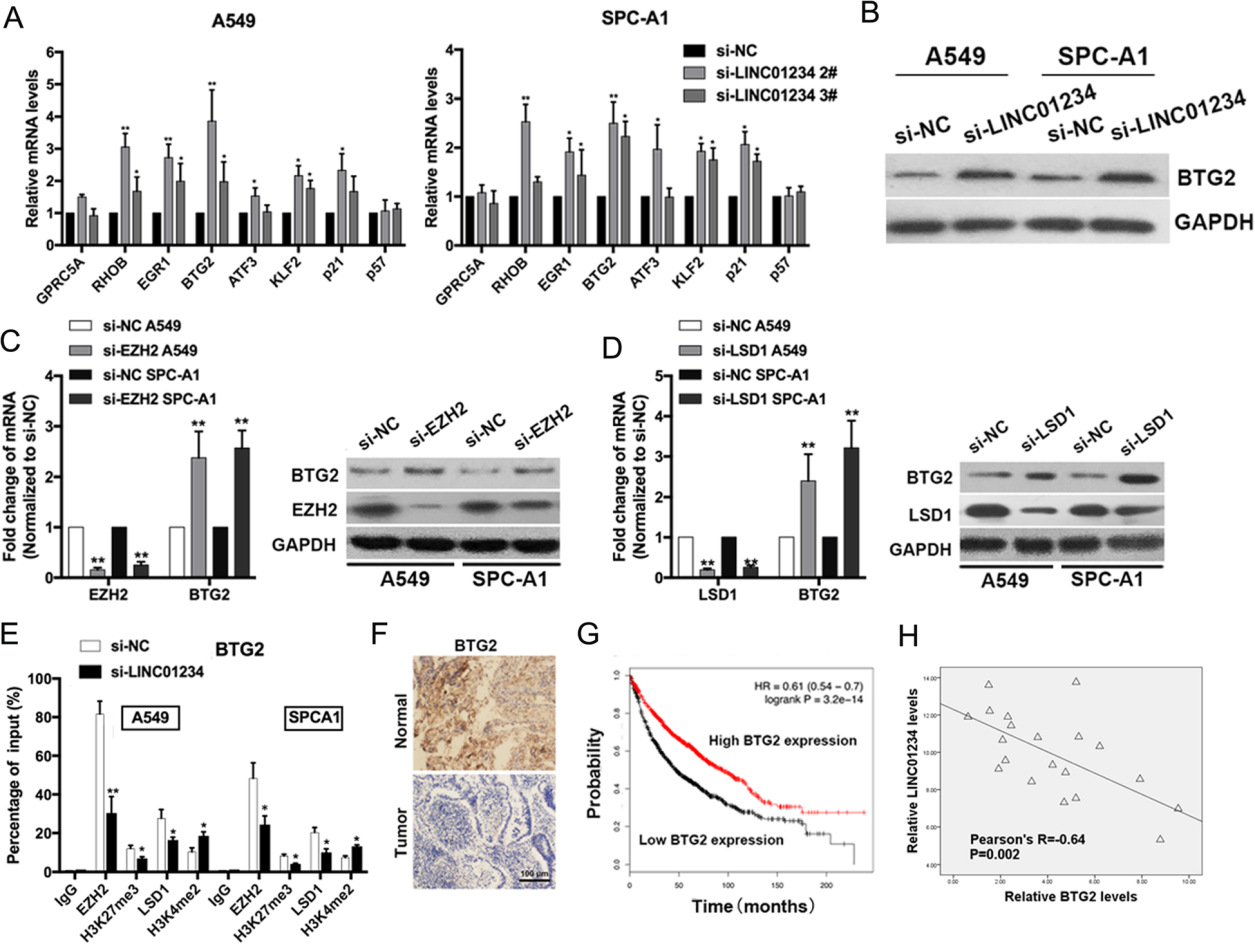
LINC01234 represses BTG2 expression by binding to EZH2 and LSD1 in NSCLC cells. **a** qRT-PCR analysis of mRNA levels of selected genes in LINC01234-depleted A549 and SPC-A1 cells compared with control cells. **b** Western blot analysis of BTG2 protein levels in A549 and SPC-A1 cells depleted of LINC01234. **c** qRT-PCR and Western blot analysis of BTG2 and EZH2 expression in A549 and SPC-A1 cells transfected with control (NC) siRNA and either EZH2 siRNA. **d** qRT-PCR and western blot analysis of BTG2 and LSD1 expression in A549 and SPC-A1 cells transfected with si-NC and either LSD1 siRNA. **e** ChIP-qPCR assay showing EZH2 and LSD1 occupancy on the BTG2 promoters and reduction by LINC01234 knockdown. **f** Immunostaining of BTG2 in NSCLC and normal tissues. **g** Kaplan–Meier survival plots of OS based on BTG2 expression in lung cancer patients (*n* = 1928). **h** Correlation analysis of LINC01234 expression and BTG2 expression. *P < 0.05, **P < 0.01

According to the RNA-seq results, we selected eight genes with altered expression involved in cancer cell migration and invasion for further study (Fig. 4a). The identified gene expression changes were validated in A549 and SPC-A1 cells by qRT-PCR. BTG2 is of particular interest because of its remarkable expression fold change upon LINC01234 knockdown (Fig. 6a). Western blot analysis showed that knockdown of LINC01234 significantly increased BTG2 protein levels in NSCLC cells (Fig. 6b), which is consistent with the qRT-PCR results (Fig. 6a) and suggests that BTG2 may play a role in mediating the functional effects of LINC01234. Our findings indicate that, in addition to acting as a ceRNA in the cytoplasm, LINC01234 may also act via EZH2 and LSD1 to regulate transcription of target genes (such as BTG2) in the nucleus. To test this hypothesis, we examined the behavior of A549 and SPCA1 cells expressing EZH2 or LSD1 siRNA. Interestingly, siRNA-mediated knockdown of the LINC01234-binding proteins EZH2 and LSD1 induced BTG2 expression at both the mRNA and protein levels (Fig. 6c, d). Importantly, ChIP assays showed that EZH2 and LSD1 bound directly to the promoter regions of BTG2 and induced deposition of the trimethylated histone 4 lysine 37 (H3K27) and demethylated H3K4 marks (Fig. 6e), which are associated with transcriptional repression. These data suggest that LINC01234 acts, at least in part, by interacting with EZH2 and LSD1, which, in turn, repress transcription of BTG2.

### The oncogenic role of LINC01234 is mediated by inhibition of BTG2 expression

Finally, we examined the potential role of BTG2 as a tumor suppressor in NSCLC. In support of this possibility, BTG2 protein was expressed at lower level in tumor tissues compared with normal tissues (Fig. 6f). Kaplan–Meier analysis of patient survival showed that BTG2 expression positively correlated with prognosis, which was supported by Kaplan–Meier Plotter analysis (www.kmplot.com) [14] (Fig. 6g). We analyzed the correlation between BTG2 and LINC01234 expression, and found that BTG2 expression in paired NSCLC tissues and normal lung tissues was negatively correlated with LINC01234 expression (Fig. 6h).

**Fig. 7:**
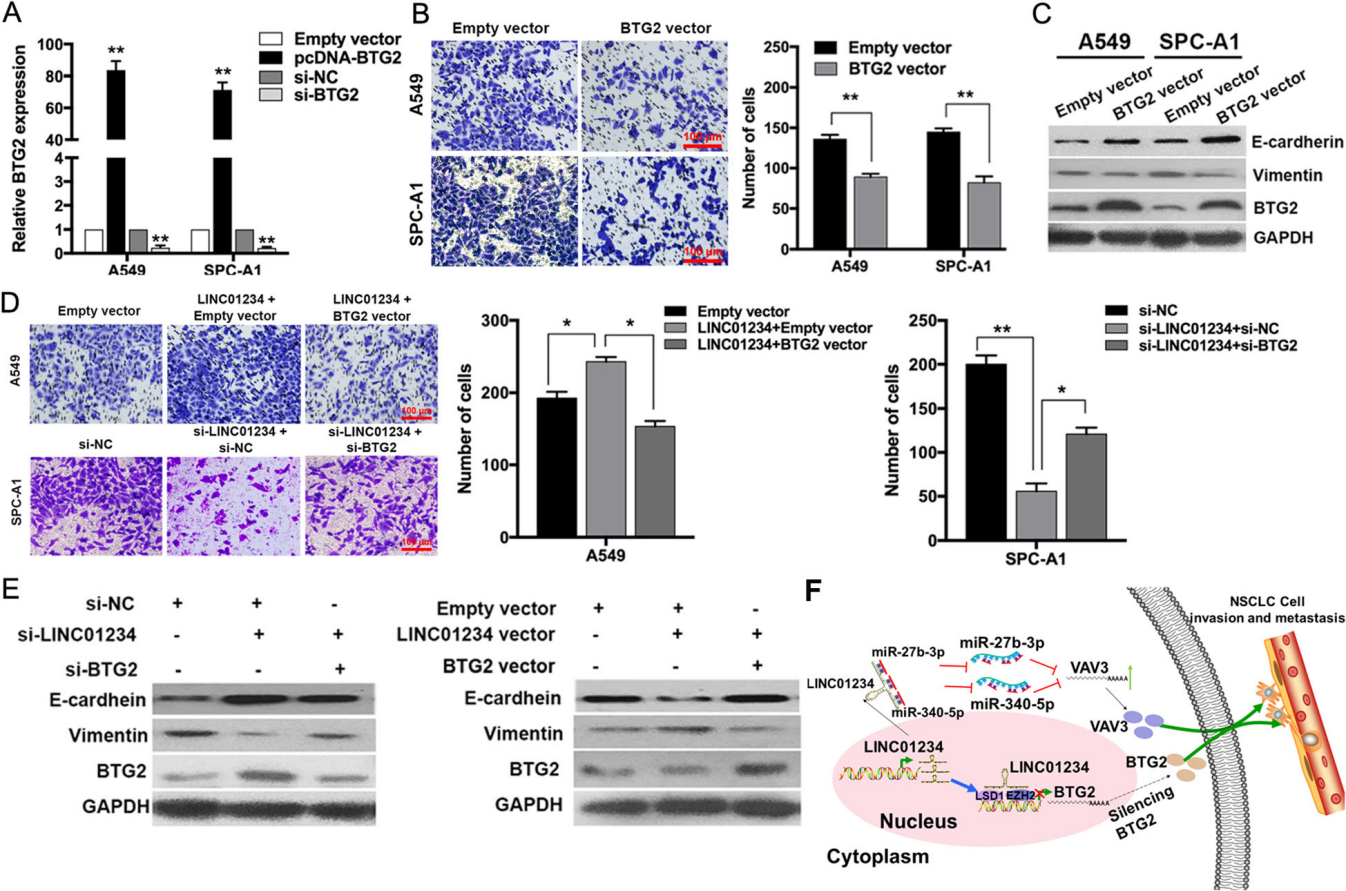
BTG2 has tumor suppressor functions in NSCLC cells. **a** qRT-PCR analysis of BTG2 mRNA levels in A549 and SPC-A1 cells overexpressing or depleted of BTG2. **b** Transwell assay of NSCLC cells overexpressing BTG2. **c** Western blot analysis of BTG2, E-cadherin and Vimentin protein levels after overexpression of BTG2. **d** Transwell assay of A549 and SPC-A1 cells overexpressing or depleted of LINC01234 and BTG2. **e** BTG2, E-cadherin and Vimentin protein levels were detected after co-transfection of cells with si-BTG2 and si-LINC01234 (left). BTG2, E-cadherin and Vimentin protein levels were detected after co-transfection of cells with BTG2 vector and LINC01234 vector (right). **f** Summary of the mechanism of LINC01234 in NSCLC cells. *P < 0.05, **P < 0.01

To better understand the biological function of BTG2, we performed gain-of-function assays. A549 and SPC-A1 cells were transfected with BTG2 overexpression vectors or siRNAs, and the mRNA and protein levels were confirmed to be significantly upregulated or downregulated by qRT-PCR and Western blotting (Fig. 7a, c). Importantly, overexpression of BTG2 led to impairment and invasion by A549 and SPC-A1 cells (Fig. 7b). Interestingly, expression of EMT marker E-cadherin was increased, whereas Vimentin was decreased, in BTG2 overexpressed cells (Fig. 7c). We next performed rescue experiments to determine whether LINC01234-mediated regulation of invasion is dependent on repression of BTG2. A549 cells were cotransfected with si-LINC01234 and si-BTG2, and SPC-A1 cells were cotransfected with LINC01234 and BTG2 vector. Indeed, co-transfection of cells with si-BTG2 partially rescued si-LINC01234-mediated impairment of invasion and EMT process. Conversely, BTG2 overexpression partially rescued the LINC01234-induced promotion of cell invasion and EMT progress (Fig. 7d, e). Taken together, these findings demonstrate that LINC01234 affects NSCLC cell development and progression through the epigenetic repression of BTG2, at least in part.

## Discussion

Multiple factors are involved in tumor progression, and numerous recent studies have implicated lncRNAs as critical regulators of these processes. In the present study, we identified a NSCLC metastasis-associated lncRNA LINC01234, which is highly expressed in metastatic NSCLC tissues and significantly associated with shorter survival time. In addition, modulation of LINC01234 expression revealed its oncogenic activity through promotion of cell migration, invasion, supporting a potential role for LINC01234 dysregulation in NSCLC progression. Indeed, complementary in vivo studies using a mouse model revealed that LINC01234 plays a key role in tumor metastasis.

A great number of recent studies have demonstrated that lncRNAs contribute to cancer progression through numerous mechanisms; for example, by recruiting histone modification enzymes (such as EZH2, SUZ12, and LSD1) that repress or activate gene transcription [15, 16] acting as competing endogenous RNAs (ceRNAs) or sponges to inhibit microRNA (miRNA) activity [11], interacting with RNA-binding proteins (e.g., STAU1, UPF1, and hnRNPL) to regulate mRNA stability [10, 17, 18] and encoding small active peptide [19]. In the present study, we investigated the molecular mechanisms through which LINC01234 regulated the tumor progression-related behavior of NSCLC cells, and found that LINC01234 interacted with several RNA-binding proteins, including Ago2, EZH2, LSD1 and SUZ12. Accumulating evidence has revealed the existence of a widespread ceRNA interaction network in which lncRNAs compete with miRNAs for binding sites in the 3′-UTR of target mRNAs. For example, HOXA11-AS promotes gastric cancer cell growth by functioning as a ceRNA for miR-1297 [16], while HOXD-AS1 acts as a ceRNA for miR-130a-3p and facilitates liver cancer metastasis by regulating SOX4 [20]. Here, we showed that LINC01234 is a ceRNA for miR-340-5p and miR-27b-3p and antagonizes their repression of VAV3 protein translation in NSCLC cells. MiR-340 and miR-27b have been reported to have tumor-suppressive functions in multiple cancers. For instance, Li et al. reported that miR-340 inhibits ovarian cancer metastasis via inactivation of NF-x03BA;B1 [21]. Yan et al. found that miRNA-340 inhibits the invasion of esophageal cancer by targeting phosphoserine aminotransferase 1 [22]. MiR-27b has also been shown to inhibit gastric cancer metastasis by targeting NR2F2 [23], and suppress NSCLC invasion by targeting SP1 [24]. In addition to these findings, our results showed that overexpression of miRNA-340 and miR-27b repressed NSCLC cell invasion by targeting VAV3 expression. The VAV family of guanine nucleotide exchange factors participates in numerous important pathological processes, including oncogenesis and cell transformation. Recent studies showed that VAV3 expression is increased in breast, prostate, and colorectal cancer [25–27], and VAV3 promoted cell metastasis in gastric cancer [28]. Consistent with this, we also found that VAV3 was upregulated in NSCLC, and its knockdown inhibited NSCLC cell invasion. These findings suggest that the LINC01234–miR-340-5p/miR-27b-3p–VAV3 axis plays important roles in the progression of NSCLC.

Our data showed that, in addition to functioning as a ceRNA in the cytoplasm, LINC01234 interacts with some well-known histone modification enzymes, such as EZH2, SUZ12, and LSD1, to repress target gene expression (BTG2) in the nucleus. EZH2 and SUZ12 are core subunits of the Polycomb repressive complex 2 (PRC2), which suppresses gene transcription by trimethylating H3K27. In human melanoma cells, loss of EZH2 partially interfered with invasion capacity [29]. LSD1, one of the first discovered protein lysine demethylases, demethylates H3K4me2 to H3K4me1 or H3K4me0 [30]. LSD1 has been found to contribute to invasion and metastasis of luminal breast cancer cells [31]. We propose that LINC01234 acts as a scaffold and recruits EZH2 and LSD1 to the promoter regions of BTG2, thereby repressing its transcription in NSCLC cells. BTG2 is a newly identified tumor suppressor that belongs to the BTG/TOB family, and many studies have revealed that BTG2 is downregulated in various cancers, including breast cancer, osteosarcoma, and bladder cancer. BTG2 inhibited the hepatocellular carcinoma cell invasion and metastasis [32–34]. Our data show that BTG2 expression is reduced in NSCLC tissues compared with normal lung tissues, and is associated with shorter patient survival. In NSCLC, overexpression of BTG2 inhibited cell invasion, and rescue experiments confirmed that the oncogenic function of LINC01234 is partly dependent on repression of BTG2 transcription.

## Conclusion

In summary, the present study identifies a NSCLC metastasis-associated lncRNA, LINC01234, which is upregulated in human NSCLC and associated with poorer prognosis. LINC01234 exerts its oncogenic function by promoting cell invasion and metastasis, which it achieves by acting as a miRNA sponge in the cytoplasm and a scaffold for histone modification enzymes in the nucleus. These findings advance our understanding of the lncRNA–miRNA–mRNA ceRNA network in NSCLC progression, and suggest that LINC01234 may have utility as a diagnostic marker and/or therapeutic target for NSCLC (Fig. 7f). However, whether LINC01234 displays similar functions, mechanisms of action, and targets in other cancers is unknown and should be investigated in the future.

## Supplementary information

**Additional file 1: Figure S1**. (A) Metastases to other organs (Kidney, liver, spleen, and intestines). (B) Distribution of LINC01234 in NSCLC tumor tissues. **Table S1**. Primer, siRNA and shRNA sequences, antibodies. **Table S2**. Correlation between LINC01234 expression and clinicopathological characteristics of NSCLC patients (*n* = 45). **Table S3**. Univariate and multivariate analysis of clinicopathological factors for over-survival in NSCLC patients (*n* = 45).

## Data Availability

The dataset(s) supporting the findings of this study are included within the article.

## Abbreviations

CeRNA: Competing endogenous RNA
lncRNA: Long noncoding RNA
NC: Negative control
ncRNA: Noncoding RNA
NSCLC: Non-small-cell lung cancer
OS: Overall survival
PFS: Progression-free survival
PRC2: Polycomb repressive complex 2
RIP: RNA immunoprecipitation
