# Supplementary material for: Up-regulated LINC01234 promotes non-small-cell lung cancer cell metastasis by activating VAV3 and repressing BTG2 expression
Source: J Hematol Oncol. 2020 Jan 20;13:7. doi: 10.1186/s13045-019-0842-2 (PMC6972004; doi:10.1186/s13045-019-0842-2)
Supplement: Supplementary file 1 — Additional file 1: Figure S1. (A) Metastases to other organs (Kidney, liver, spleen, and intestines). (B) Distribution of LINC01234 in NSCLC tumor tissues. Table S1. Primer, siRNA and shRNA sequences, antibodies. Table S2. Correlation between LINC01234 expression and clinicopathological characteristics of NSCLC patients (n = 45). Table S3. Univariate and multivariate analysis of clinicopathological factors for over-survival in NSCLC patients (n = 45). [file 13045_2019_842_MOESM1_ESM.zip › Table S2 and Table S3.docx]

**Table S2.** Correlation between LINC01234 expression and clinicopathological characteristics of NSCLC patients (n = 45)

| **Characteristics** | **LINC01234** | | **P** |
| --- | --- | --- | --- |
|  | **Low no. cases (%)** | **High no. case (%)** | **Chi-squared test**  **P-value** |
| **Age (years)** | | | |
| >65 | 12 | 15 | 0.465 |
| ≤65 | 10 | 8 |  |
| **Gender** | | | |
| Male | 13 | 13 | 0.862 |
| Female | 9 | 10 |  |
| **Smoking history** | | | |
| Smokers | 14 | 12 | 0.436 |
| Never smokers | 8 | 11 |  |
| **Histological subtype** | | | |
| Squamous cell carcinoma | 10 | 10 | 0.894 |
| Adenocarcinoma | 12 | 13 |  |
| **Histologic grade** | | | |
| Well differentiated | 8 | 6 | 0.706 |
| Moderately differentiated | 6 | 7 |  |
| Poorly differentiated | 8 | 9 |  |
| Undifferentiated | 0 | 1 |  |
| **Tumor size** | | | |
| ≤5cm | 17 | 11 | 0.042^*^ |
| >5cm | 5 | 12 |  |
| **Lymph node metastasis** | | | |
| Negative | 15 | 7 | 0.011^*^ |
| Positive | 7 | 16 |  |
| **TNM Stage** | | | |
| Ia + Ib | 8 | 1 | 0.015^*^ |
| IIa + IIb | 9 | 10 |  |
| IIIa | 5 | 12 |  |

*P<0.05 was considered significant

**Table S3.** Univariate and multivariate analysis of clinicopathological factors for over-survival in NSCLC patients (n = 45)

| Variables | Univariate analysis | | | Multivariate analysis | | | |
| --- | --- | --- | --- | --- | --- | --- | --- |
|  | HR | 95% CI | p value | | HR | 95% CI | p value |
| Age  (≤65/>65) | 1.183 | 0.516-2.708 | 0.691 |  | |  |  |
| Gender  (Male/Female) | 1.919 | 0.858-4.291 | 0.112 |  | |  |  |
| Smoking history  (no vs yes)  Histological subtype  (LUAD /LUSC) | 1.442  1.293 | 0.655-3.175  0.585-2.86 | 0.363  0.525 |  | |  |  |
| Histologic grade  (Well, mod / Poor, undi) | 2.243 | 1.374-3.659 | 0.001* | 1.622 | | 0.962-2.734 | 0.070 |
| Tumor size  (≤5/>5) | 2.181 | 0.984-4.831 | 0.055 |  | |  |  |
| Lymphatic metastasis  (no vs yes) | 6.072 | 2.076-17.764 | 0.001* | 2.053 | | 0.511-8.254 | 0.311 |
| TNM stage  (III+IV vs I+II) | 3.093 | 1.471-6.501 | 0.003* | 1.456 | | 0.582-3.687 | 0.418 |
| LINC01234 expression  (high vs low) | 4.749 | 1.781-12.665 | 0.002* | 2.931 | | 1.027-8.363 | 0.044* |

HR, hazard ratio; 95 % CI, 95 % confidence interval, * Overall P < 0.05.
