# Supplementary figures and images for: Up-regulated LINC01234 promotes non-small-cell lung cancer cell metastasis by activating VAV3 and repressing BTG2 expression
Source: J Hematol Oncol. 2020 Jan 20;13:7. doi: 10.1186/s13045-019-0842-2 (PMC6972004; doi:10.1186/s13045-019-0842-2)

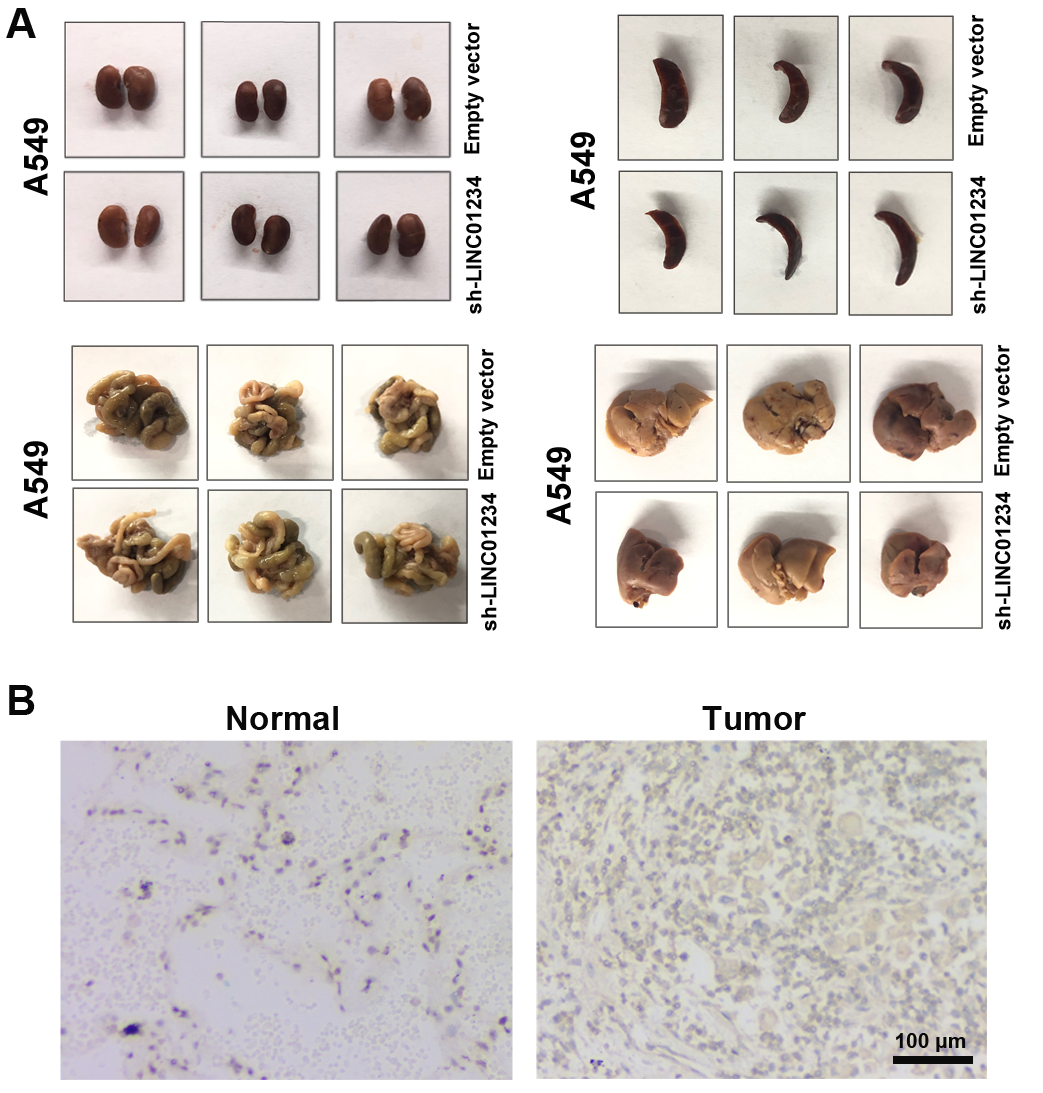

Supplement: Supplementary file 1 — Additional file 1: Figure S1. (A) Metastases to other organs (Kidney, liver, spleen, and intestines). (B) Distribution of LINC01234 in NSCLC tumor tissues. Table S1. Primer, siRNA and shRNA sequences, antibodies. Table S2. Correlation between LINC01234 expression and clinicopathological characteristics of NSCLC patients (n = 45). Table S3. Univariate and multivariate analysis of clinicopathological factors for over-survival in NSCLC patients (n = 45). [file 13045_2019_842_MOESM1_ESM.zip › Figure S1.tif]
